# Microglia and astrocyte involvement in neurodegeneration and brain cancer

**DOI:** 10.1186/s12974-021-02355-0

**Published:** 2021-12-23

**Authors:** Arthur A. Vandenbark, Halina Offner, Szymon Matejuk, Agata Matejuk

**Affiliations:** 1 Neuroimmunology Research, R&D-31, VA Portland Health Care System, 3710 SW U.S. Veterans Hospital Rd., Portland, OR 97239 USA; 2 Department of Neurology, Oregon Health & Science University, 3181 SW Sam Jackson Park Rd., Portland, OR 97239 USA; 3 Department of Molecular Microbiology and Immunology, Oregon Health & Science University, 3181 SW Sam Jackson Park Rd., Portland, OR 97239 USA; 4 Department of Anesthesiology and Perioperative Medicine, Oregon Health & Science University, 3181 SW Sam Jackson Park Rd., Portland, OR 97239 USA; 5 Medical Student of Jagiellonian University, Cracow, Poland; 6 Department of Immunology, Collegium Medicum, University of Zielona Góra, Zielona Góra, Poland

**Keywords:** Microglia, Astrocytes, CNS, Neuroinflammation, Neurodegeneration, Alzheimer disease, FTLD, Astrocytoma

## Abstract

The brain is unique and the most complex organ of the body, containing neurons and several types of glial cells of different origins and properties that protect and ensure normal brain structure and function. Neurological disorders are the result of a failure of the nervous system multifaceted cellular networks. Although great progress has been made in the understanding of glia involvement in neuropathology, therapeutic outcomes are still not satisfactory. Here, we discuss recent perspectives on the role of microglia and astrocytes in neurological disorders, including the two most common neurodegenerative conditions, Alzheimer disease and progranulin-related frontotemporal lobar dementia, as well as astrocytoma brain tumors. We emphasize key factors of microglia and astrocytic biology such as the highly heterogeneic glial nature strongly dependent on the environment, genetic factors that predispose to certain pathologies and glia senescence that inevitably changes the CNS landscape. Our understanding of diverse glial contributions to neurological diseases can lead advances in glial biology and their functional recovery after CNS malfunction.

## Introduction

Brain homeostasis is based on the interplay among all cell types, with microglia and astrocytes subserving a wide array of salient neuronal functions [7]. In their resting state, microglia display ramified morphology and surveying properties. During development, microglia actively communicate with other brain cells, contributing to neurogenesis and synaptic pruning. In the adult brain, they participate in neuromodulation, surveillance and monitoring, synaptic plasticity, learning and memory [82]. Astrocytes, the most numerous cells in the mammalian brain, stay in close contact with all CNS-resident cells and occupy strategic locations including blood vessels. During brain development, astrocytes synchronize synapse growth and modulate neuronal circuity [3, 15]. In the adult brain they are responsible for the maintenance of blood–brain barrier (BBB) integrity and metabolic coupling, ion buffering, neurotransmitter homeostasis, production of neuroactive factors (ATP, TNF-α) and control of neuronal synchronization and proper functioning of synaptic circuits [110]. Beyond homeostatic function, microglia and astrocytes are able to participate in inflammatory responses by taking on the role of local immune cells. During any disturbance or loss of homeostasis, microglia become activated, change their morphology and phenotype and increase their motility and phagocytic abilities [49]. Astrocytic responses to environmental changes include hypertrophic morphology, upregulation of GFAP, scar formation, variations in intracellular Ca^2+^ levels, activation of purinoreceptors and production of the inflammatory cytokines, NO and ROS [22, 87]. Similar to microglia, the activated state of astrocytes is a complex phenomenon recently revisited and described by Escartin et al. [21].

Both types of cells are equipped with a set of damage sensors, receptors and inflammatory mediators capable of responding to various brain injuries such as infection, stroke, traumatic brain injury, neurodegeneration and cancer. Although the ultimate goal of glial-mediated neuroinflammation aims at elimination of the threat, glial scar formation, resolving the occurring injury and restoring brain homeostasis, the results of such responses are not always beneficial, especially in the context of neurodegeneration, where chronic exposure to macrophage inflammatory stimuli can induce neurotoxic reactive astrocytes [53]. Whether glial cells adopt a phenotype that aggravates tissue injury or promotes brain repair depends on a basic set of factors, including the nature of the damaging element (toxic protein vs. LPS, acute injury vs. neurodegeneration), severity of injury, precise constellation of signals from the environment, presence of antagonistic mediators, current activation status of other cells (macrophage infiltrates) and the concentration of immunological mediators. For example, one of the factors that controls microglial activity is S100B. Astrocytes under normal conditions constitutively release S100B, which acts as a neurotrophic factor, protecting microglia from neurotoxins. However, high concentrations of S100B bind the receptor for advanced glycation end products (RAGE) leading to microglial activation. In addition to the production of the same mediators, each cell type can produce specific factors that act on the partner or on distant cells (e.g., astrocytes can secrete granulocyte and granulocyte–macrophage colony-stimulating factors to induce migration of granulocytes and macrophages to the CNS). Finally, the response largely depends on the disease context, brain area and disease stage. One example is loss of astrocytic domain organization in almost all neurological disorders, a phenomenon that is not observed in Alzheimer disease (AD).

During the past decades, several clinical trials using agents that counteract β-amyloid (Aβ) deposition in AD have failed. Current therapies counteracting the immune responses both in neurodegenerative conditions and cancer are too generic and may not sufficiently account for specific glial involvement. Research into glial biology is rapidly growing with the hope of finding new therapies for glial-specific targets. In this review we will attempt to address the complex issue on astrocyte and microglial cross-talk in the pathogenesis of the two most common neurodegenerative disorders, AD and progranulin-related frontotemporal lobar dementia **(**GRN-FTLD), as well as astrocytoma. A better understanding of microglia and astrocyte interactions may aid in the discovery and use of glia-based therapies for functional repair.

### Microglia and astrocytes in Alzheimer disease

Alzheimer disease is the most common neurodegenerative disease with the deposition of extracellular amyloid-β (Aβ) proteins and neuronal intracellular neurofibrillary tangles (NFT) comprising hyperphosphorylated tau proteins. The depositions of Aβ and NFT are primarily observed in entorhinal cortex and “spread” to the hippocampus and cerebral cortex as the disease progresses leading to neuronal loss, dementia and alterations in glial cells. Genetic, epigenetic and environmental factors play a role in the pathogenesis of AD [1]. There are two main and distinctive forms of the disease, early and late-onset AD. Early onset is a familial form caused by mutations in the genes responsible for Aβ production and degradation. These genes encode Aβ precursor protein (APP), presenilin 1 (PSEN1) and presenilin 2 (PSEN2), the latter two representing β- and γ-secretases involved in the cleavage of APP [77]. Most cases of AD are sporadic late-onset cases involving environment risk factors and many genes associated with the immune response, cholesterol metabolism and regulation of endocytosis [77]. The core transcriptional features of cellular identity via integrative gene coexpression analysis of intact tissue samples revealed that the expression levels of APP and PSEN1 genes correlated with the variability of neuronal and oligodendroglial abundance, respectively [43]. In contrast, late-onset AD showed increased expression levels of apolipoprotein E (APOE) and Triggering receptor expressed on myeloid cells 2 (TREM2) that correlated, respectively, with the abundance of astrocytes and microglia, as well as with age and neurodegeneration.

#### Communication among neurons, microglia and astrocytes in AD

**Fig. 1:**
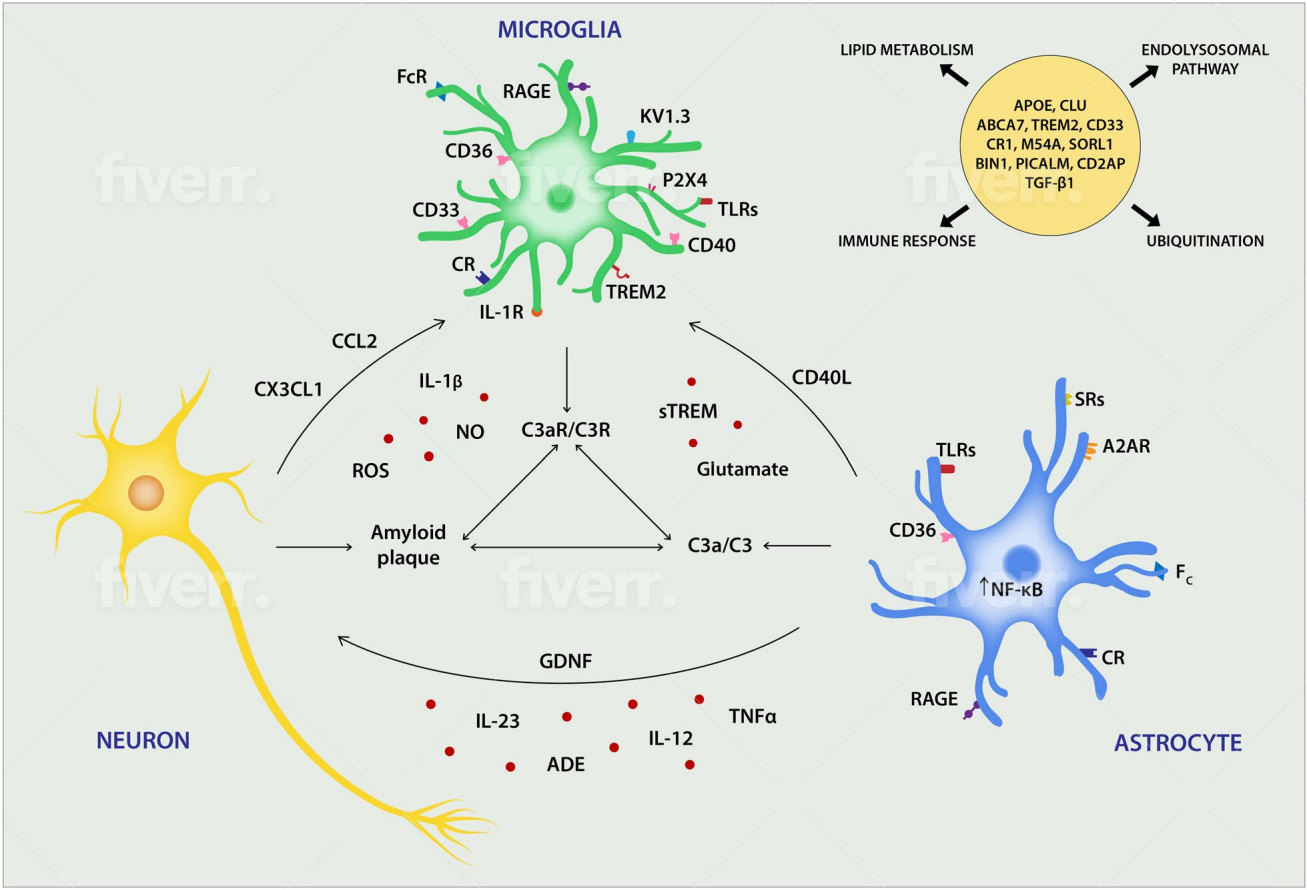
Three-party cross-talk among neurons, microglia and astrocytes in AD. Microglia and astrocytes actively respond to changes in the environment caused by amyloid (Aβ) depositions. Glia change their morphology and function, release neurotransmitters, immunomodulators such as cytokines, chemokines, complement factors and modulate each other’s activity as well as activity of other cells in the environment. Evident communication in AD is mediated by the neuronal Aβ-astrocytic C3/C3a–microglial C3aR axis. Some molecules and receptors as well as some genes and molecular pathways participating in the pathogenesis of the disease are shown. *ADE* astrocyte-derived exosomes, *GDNF* glial-derived neurotrophic factor, *RAGE* receptor for advanced glycation end products, *sTREM* soluble triggering receptor expressed on myeloid cells

Microglia and astrocytes react to changes in their environment, so it is not surprising to find them in their activated forms around amyloid plaques in AD. Aβ mainly produced by neurons activate microglia and astrocytes to capture and clear it from the brain to save neurons. Protective mechanisms that occur during early stages of disease include phagocytosis of Aβ, release of neuroprotective cytokines, exosomes, neurotrophic factors and neurotransmitters. Primary neurons disturbed by amyloid communicate with microglia via CX3CL1 (fractalkine) and CCL2 using the glial-derived neurotrophic factor (GDNF) released by astrocytes (Fig. 1). The intercellular cross-talk among neurons, astrocytes and microglia is mediated by the neuronal Aβ-astrocytic C3/C3a–microglial C3aR axis. The overproduction of Aβ by neurons stimulates astroglial NF-κB that induces expression and secretion of C3 [51]. In vitro and in vivo studies show that astrocytic C3 interacts with the microglial C3a receptor to induce microglial Aβ phagocytosis and exacerbation of Aβ pathology [51]. Astrocyte-derived exosomes (ADE) are rich in complement compounds like C1q, C4b, C3d, factor B, factor D, Bb, C3b, and C5b–C9 [28]. Activated astrocytes and microglia can respond to amyloid depositions directly by expressing scavenger receptors (SRs), Fc receptors, complement receptors (CR), RAGE, CD36 and TLRs. CD36 facilitates recruitment of microglia to plaque depositions and promotes inflammation initiated by a TLR4/6 heterodimer [89]. Other TLRs (2, 3, 7 and 8) play pivotal roles in modulating neurodegenerative pathways, inducing activation and release of TNF-α, NO and superoxide [95]. TNF-α, one of the immune genes upregulated during the progression of AD, is essential and sufficient for induction of neurotoxic astrocytes and thus is an integral component of neuropathological changes. Furthermore, reactive microglia stimulate tau pathology in a cell-autonomous manner [58], forming a barrier that impacts plaque composition and toxicity [16]. It is noteworthy that the spread of tau deposition can be reduced by treatment with an interleukin 1 receptor antagonist [58].

Reactive astrocytes surround Aβ plaques and express receptors such as RAGE and SRs mentioned above, as well as a low-density lipoprotein receptor-like protein and membrane-associated proteoglycans that are known to bind Aβ. Reactive astrocytes form the border between the focal lesion and the surrounding CNS tissue and degrade amyloid plaques in an ApoE-dependent manner [46]. Increased plaque load has been found in GFAP-Vim-mice lacking astrocytes crossed with a mouse model of AD-like pathology [74]. In astrocytes, ApoE is not only involved in cholesterol metabolism, but also participates in synapse pruning, and therefore malfunction of ApoE in AD can cause fatal consequences for circuit function. ApoE is mainly expressed by astrocytes and reactive microglia. In humans, there are three genetic isoforms of apolipoprotein E responsible for cholesterol transport, and one of them, ApoE4, is the best known risk factor for AD [55]. Generally, lipid metabolism is highly associated with AD pathology. Lipid transport is partially controlled by clusterin (CLU), a gene that also mediates apoptosis and an immune response. CLU in conjunction with ApoE is thought to be upregulated to protect neurons during damage mediated through astrocytes and microglia, as inferred from an increased plaque load in double ApoE and CLU knock-out APP transgenic mice [77]. In humans, loss-of function alleles for another gene linked to lipid transport, an ATP-binding cassette transporter A7 (ABCA7), also a genetic risk factor for AD), has been associated with cortical and hippocampal atrophy [77]. It has been shown that stimulation of liver X receptor alpha (LXRa), a cholesterol sensor that regulates the function of immune cells and cholesterol metabolism, leads to increased levels of ApoE in astrocytes and increased phagocytosis of fibrillar Aβ by microglia [94]. The protective role of microglia and astrocytes ends when the immune response becomes chronic, and the phagocytic capacity of glia becomes overwhelmed. Hyperstimulation of glial cells can be fueled by various channel activators like Kv1.3 and P2X4 in microglia [69]. Further, release of various neuroinflammatory factors including IL-1β, TNF-α, NO, ROS, and neurotoxic products like glutamate lead to excitotoxicity, unbalanced synaptic engulfment and eventually neuronal death.

#### Subsets of reactive microglia and astrocytes in AD: a lesson from single-cell sequencing

Recently, exciting single-cell and nuclei sequencing discoveries revealed many different subsets of responsive microglia and astrocytes in AD. Glial activation and subsequent inflammatory events are key contributors to the pathogenesis of AD and not simply a response to amyloid deposition. To this point, Mathys et al. described successive stages of microglial changes and the implementation of the neurodegenerative reprogramming that included increased proliferation followed by upregulation of MHC class II and antiviral and interferon-response genes [63]. Two distinct reactive microglia subpopulations were identified at a later stage in a mouse model of neurodegeneration based on expression of Type I or type II interferon-response genes. Both types of interferon-response genes are actively involved in immune responses but how these particular microglia subpopulations contribute to neurodegeneration remains to be addressed. Similarly, microglia with upregulated expression of both IFN-γ responsive and MHC II genes showing a fully inflammatory response were found in EAE, an animal model for MS [68]. In this study a combination of high-dimensional single-cell cytometry and fate mapping was used to create a murine immune cell atlas for studying immune responses in the brain, particularly during aging, neurodegeneration (models of AD) and neuroinflammation (MS). In EAE, microglia were found to be homogenously reactive, whereas during aging and AD, responsive microglia constituted only a small subpopulation. Using the same, single-cell RNA sequencing technology, a new subset of microglia cells present in animal models of AD, amyotrophic lateral sclerosis (ALS) and aging, called disease-associated microglia (DAM), was identified as part of microglia sensory mechanism for damage detection [44]. DAMs are characterized by downregulation of microglial homeostatic genes including purinergic receptors, CX3CR1 and Tmem119, and upregulation of genes related to phagocytosis and lipid metabolism as well as expression of many genes that are known AD risk factors including Apoe, Tyrobp, Ctsd, Lpl and TREM2. The TREM/Tyrobp (DAP12) signaling pathway is critical for reduction of Aβ deposition and limitation of neurodegeneration by dampening inflammatory responses in microglia via reducing cytokine production and increasing phagocytic activity. As found in different AD mouse models, TREM2 is necessary for full activation of the DAM program. It postulated that amyloid plaques in the early stage of the disease activate DAMs, which are fully functional in protecting against disease. However, over time, plaques accumulate and stimulate the inflammasome in microglia leading to disease progression [36].

The first in vivo comparison of mouse and human CNS microglia heterogeneity at a single-cell resolution confirmed the existence of developmental heterogeneity of microglia and significant regional differences in the brain, as well as disease-associated patterns. However, there were high inter-individual variations in both mice and humans [61]. In that study, mouse embryonic microglia were characterized by increased expression of genes related to lysosomal activity, such as Ctsb or Lamp1, but also apolipoprotein E (ApoE). Moreover, the postnatal brains displayed high expression of Sparc and Cst3 encoding cystatin C, which is involved in CNS neurodegenerative diseases. The same Cst3 gene was also detected in a subpopulation of ALDH1L1 + astrocytes in the adult mouse brain. The most uniform phenotype of existing microglia was found in adult brains from both mice and humans. Homeostatic human microglia only partially overlap with those of adult mouse microglia. Analysis of human MS brains revealed high inter-individual heterogeneity and similarity to some subtypes of microglia in disease models. Interestingly, when distorted, microglia were able to quickly recover and transform their phenotypes, confirming their remarkable plasticity to environmental changes. The microglial recovery-associated gene signature was found in the unilateral facial nerve axotomy (FNX) model of acute neurodegeneration not driven by any susceptibility gene [93]. During the onset of recovery in situ, a transient microglia subset of the facial nucleus has been identified with high expression of Apoe and Ccl5, showing lesion-dependent gene regulation [93]. The functional role of the recovery microglial subtype is presently unclear. Using the deep single-cell RNA-seq technique, Li et al. confirmed the previous finding that regardless of the region of the mouse brain, the adult homeostatic microglia displayed limited transcriptomic heterogeneity in contrast to postnatal brain microglia that were characterized by developmental complexity [50]. A new subpopulation of metabolically active microglia (WAMs), transiently present in the first postnatal week in the white matter, displayed gene expression patterns mimicking DAMs. WAMs were found to be involved in phagocytosis of newborn oligodendrocytes and most likely astrocytes. In contrast to DAMs, WAM appearance did not depend on the TREM2–APOE axis.

Human studies by Mathys et al. showed a heterogenous response to AD pathological progression between cell types, especially early in disease development [64]. Late AD pathology, however, was characterized by non-cell specific upregulation of autophagy, apoptosis and stress response genes associated with maintenance of protein integrity as a global stress response. In general, human AD pathology in microglia correlated with enrichment in immune/inflammatory pathways, Aβ clearance and genetic risk factors for AD such as TREM2, APOE and the MHC class II genes. Disease-associated signatures of astrocytes revealed reactive astrocytes with preferential expression of GLUL and the AD risk gene CLU. Recent studies by Habib et al. using single-nucleus RNA sequencing identified disease-associated astrocytes (DAAs) in the AD mouse model, but also in aged human brain. These DAAs were characterized by upregulated expression of genes involved in development, differentiation and immune responses and included encoding proteins involved in amyloid metabolism and clearance, such as CLU, Serpina3n, Cathepsin B, APOE (the latter two among genes shared with DAMs), [32]. The authors proposed a scenario of continuous DAAs activation. The process starts with a beneficial reaction called gliosis, which protects the healthy neurons. Afterwards, as a consequence of cross-talk with environmental mediators and other cells like microglia, there is continuous activation of the inflammatory response and neurotoxic factors like SerpinA3N leading to the progression of disease. Of note, the presence of a pool of genes shared between DAMs and DAAs might suggest that there is a general transcriptional program initiated in different cells in response to pathology. The most recent human study by Srinivasan et al. showed lack of DAM response in human AD microglia (HAM), and instead, HAMs exhibited accelerated aging and age-independent changes such as upregulation of APOE [88]. Surprisingly, the reduction of a homeostatic gene signature defined in mouse microglia was not observed in human samples.

The powerful sequencing technologies give us a unique opportunity to decipher the bidirectional communication mechanisms between microglia and astrocytes in many aspects of brain pathophysiology. However, they possess many technical variations and imperfections like under-sampling and bias of cells that are sequenced. It must also be noted that human “healthy” tissue, used as a control in some studies, was derived from epilepsy surgery, where multiple seizures per day are taking place. Thus, caution should be taken in interpretation of these large amounts of data. Moreover, the expression signatures found in human samples only partially overlapped those found in mice. Animal models for AD characterized by accelerated amyloid or tau proteinopathy, although very useful, do not mirror human pathology, which in most cases develops slowly with age. For example, single-cell RNA sequencing analysis of living microglia isolated from the aging and AD human cerebral cortex showed divergent enrichment for genes related to AD, in which DAM genes (in contrast to mice) were found to be present in several different human microglia subsets [71]. The complement component C1qB and the pattern recognition receptor CD14 were exclusively found in human studies [64]. Thus further single-cell resolution studies are needed to highlight the complexity of glial involvement in human AD pathology. Most importantly, it is essential to understand how these different subsets of reactive glia translate into specific functions in health but also in particular disease contexts.

#### Genetic studies confirm the glial contribution to neuroinflammation in AD

Recent genetic studies have revealed more than 30 chromosomal loci related to AD, many of which lie in non-coding regions [77]. Studies that identify single nucleotide polymorphisms in inflammatory genes associated with AD risk underline the involvement of inflammation, microgliosis and astrogliosis (Fig. 1). Inflammatory reactions that precede amyloid depositions were characterized by an increase in the level of cytokines, chemokines and complement as well as the involvement of microglia and astrocytes. It was reported that a genetic polymorphism in TGFβ1, an immunosuppressive cytokine that controls the activation of microglia, is associated with the risk of developing AD [56]. Another example of the glial participation in AD immune response is the involvement of CR1 and CD33 genes, which are also genetic risk factors for AD [84, 118]. CD33 is a member of the SIGLEC (sialic acid-binding immunoglobulin-like lectin) family of lectins able to inhibit cell signaling. CD33 was found to be upregulated in AD and the amount of amyloid depositions correlated with CD33-positive microglia in AD patient brains [29]. CD33 influences the function of TREM2, which is expressed by microglia and infiltrating monocytes and upregulated by injury in AD [67]. TREM2 binds phosphatidylserine on apoptotic cells and promotes phagocytosis of apoptotic cells and debris. It binds lipoprotein (e.g., ApoE) which in AD forms complexes with Aβ residues facilitating their removal by microglia [94]. The TREM2–APOE pathway is a major regulator of microglia function in neurodegeneration [47]. Besides phagocytosis, TREM2 also supports metabolism in microglia through mTOR signaling, and therefore, abnormal action and deficiency of TREM2 may have detrimental effects on energetic and anabolic microglial metabolism. One of the proposed therapeutic interventions in AD is based on nourishing microglial metabolism [97]. In TREM2 deficient 5XFAD mice, the total number of microglia was lower and microglia were less effective in Aβ amyloid internalization [107]. Also in humans, a homozygous loss-of-function mutation in TREM2 has been found to be associated with increased risk for AD [30]. In both humans and mice, dysfunctional TREM2 resulted in the accumulation of autophagic vesicles and increased plaque-associated neurite dystrophy [97]. Gene profiling of human astrocytes from post-mortem AD tissue revealed abnormal expression of 32 genes associated with Ca2^+^ signaling and homeostasis [103]. Reactive astrocytes in AD display spontaneous Ca2^+^ oscillations and aberrant intracellular Ca2^+^ waves [104]. As noted above, ApoE4 isoform produced by astrocytes is one of the strongest genetic risk factors of AD [55]. Recent studies demonstrated that astrocytic ApoE4 converts neuronal tau to a more aggressive state in different in vitro and in vivo tau models of AD [40]. Several studies of disease-susceptibility genes in AD revealed new candidates as disease risk factors with rare coding variants, also further confirming involvement of glia cells in the pathogenesis of this disease [86, 91].

#### Age as strongest risk factor for sporadic AD

One of the greatest risks for sporadic AD is aging, characterized by gradual loss of physiological function and cell homeostasis, decreased levels of ATP, dysregulated apoptosis, increased radical production and decrease of BBB integrity. Gradual increase of inflammation occurring also in the CNS is a key factor of aging called “inflammaging” where the immune system undergoes a process of senescence [17]. Microglia and astrocytes effected by age are less effective in the regulation of synaptic plasticity and display altered lysosomal and mitochondrial functions and become activated by environmental changes in aged brain. Aging significantly impairs cognitive properties that are severely impaired in AD patients. Transcriptomic profile of aging microglia revealed upregulated transcripts linked to brain inflammation, cell stress response and age-related diseases such as AD [9, 72]. Transcriptome-wide studies of bulk ex vivo human microglia allowed creation of an atlas of gene sets from aged humans that are mainly expressed by microglia and are associated with neuropathological tissues and susceptibility genes of AD, confirming the strong relationship between age and neurodegenerative diseases [72]. Aged human microglia were characterized by downregulated genes within the TGF-β pathway suggesting loss of homeostatic programs and induction of a reactive profile. Intriguingly, the APOEε2 haplotype was found to be associated with a reduced aging human microglia signature. However, the much stronger AD risk factor, APOEε4, has not yet been related to an aged microglia signature. Further studies of this group on aged human samples by single-cell sequencing confirmed the enrichment of microglia subsets involved in interferon response and antigen presentation, as well as genes involved in neurodegenerative diseases with only one particular subtype of microglia that has been changed (reduced) in AD [71]. In contrast to mouse models, human genes linked to DAMs or interferon-response genes were distributed across different human clusters. As shown by Hammond et al., during aging in mice, there is progressive expansion of clusters that contain very few cells in adult samples. Specifically, two microglia clusters were expanded in aged mice, one expressing a number of inflammatory signals such as CCL4 and IL-1β and the other, interferon-response genes that can modulate inflammation [34]. A phenotypic signature in a subset of microglia located around Aβ plaques in APP/PS1 mice resembled CD11c + microglia in geriatric mice, both subsets being characterized by increased phagocytosis-associated markers CD11c and CD14 [68].

Similar to microglia, aged astrocytes in mice display upregulation of inflammation-related genes but also an increase in oxidative stress genes [32]. As recently shown, old astrocytes were dysfunctional in ion buffering and glutamate clearance that impact synaptic plasticity and cognitive decline in the senescent brain [81] One suggested therapeutic strategy for memory enhancement in AD patients is to target the A2A receptor on astrocytes that is overexpressed during the disease. Ablation of this receptor in aging mice resulted in upregulation of Arc/Arg3.1, an immediate early gene that is required for long-term memory, and improved memory in mice [73]. Taken together, the changes that occur in old glia may impact the development and progression of age-related diseases such as AD.

### Progranulin-related frontotemporal lobar dementia (GRN-FTLD): a monogenic microglial disorder

**Fig. 2:**
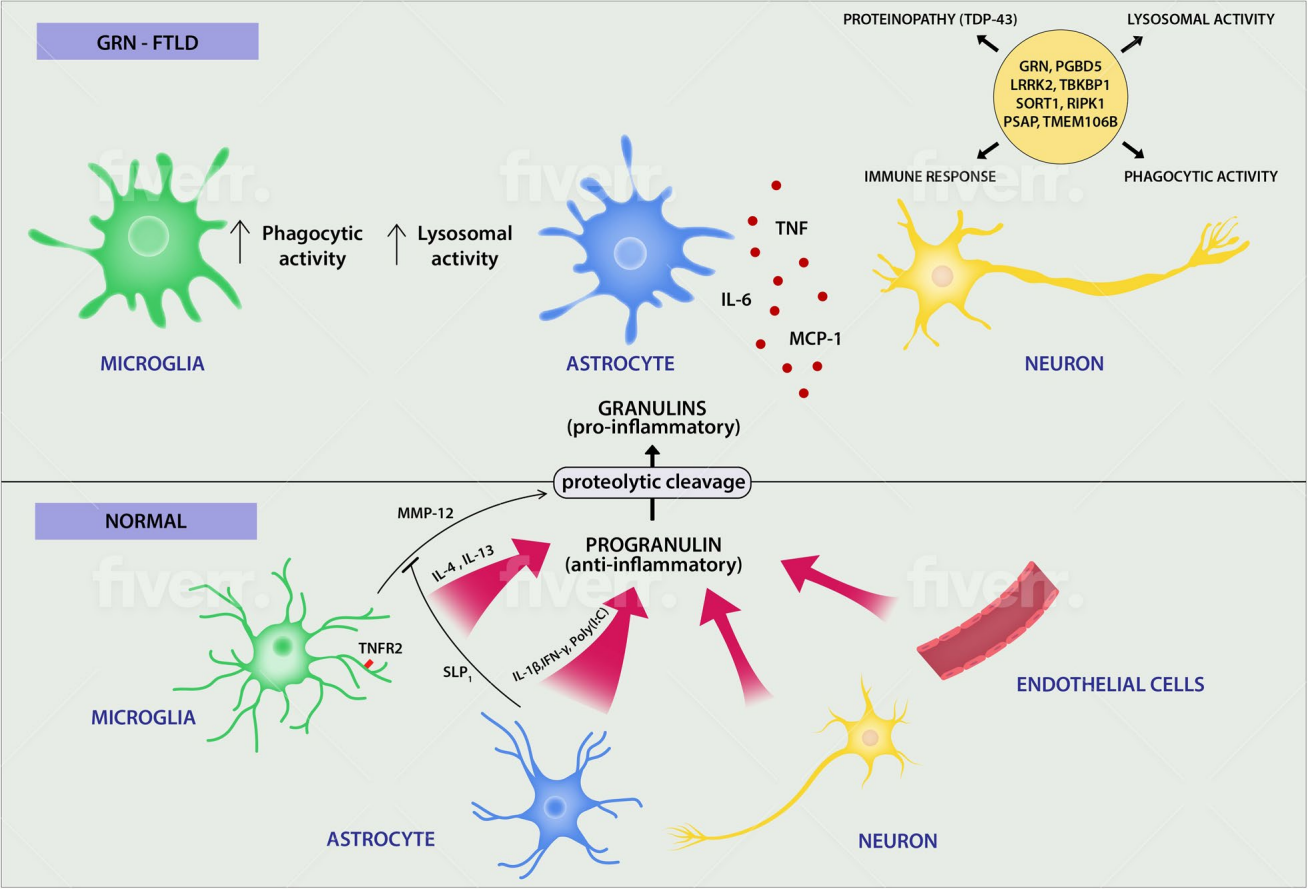
Contributions of microglia and astrocytes in the pathogenesis of GRN-FTLD. In normal conditions progranulin, an anti-inflammatory molecule with many biological functions is produced in the CNS predominantly by microglia and to a lesser degree by astrocytes, endothelial cell and neurons. Its production is differentially regulated dependent on the cell type. GRN can be degraded by microglial MMP-12 to granulins with pro-inflammatory actions. This process is regulated by astrocytic SLP1 molecule. In GRN-FTLD the inflammatory milieu driven by genetic factors and proinflammatory mediators like granulins changes the morphology and function of glia cells. Microglia and astrocytes become activated with higher lysosomal and phagocytic activities that increases TDP-43 proteinopathy. Some molecules, genes and molecular pathways participating in the process are presented. *Poly I:C* polyinosinic:polycytidylic acid, *SLP1* secretory leukocyte protease inhibitor

Frontotemporal lobar dementia (FTLD) is the second most common neurodegenerative disorder that usually occurs in middle-aged humans. The disease affects the frontal and temporal lobes with early onset of dementia and impairment of behavior, language and cognition. Like Alzheimer disease, most FTLDs are sporadic and most likely are caused by a combination of genetic and environmental factors. Some cases, however, are induced by genetic factors and are inherited in an autosomal dominant way. The disease has a large familial component, with about 30–50% of cases reporting family history of disease. The disease is associated with multiple genes (Fig. 2). Mutations in the gene that encodes progranulin (*GRN*) on chromosome 17q21–22, have been identified in patients with inherited FTLD characterized by tau-negative, ubiquitin-positive inclusions [99]. The 43 kDa transactivating DNA binding protein (TDP-43) is the major ubiquitinated protein causing proteinopathy in most cases of FTLD. Pathologic TDP-43 has been also found in ALS, 25–50% of Alzheimer’s cases, and Parkinson’s disease [4, 11, 76]. GRN mutation accounts for approximately 20% of familial and 5% of sporadic cases and results in loss of function or significantly diminished expression [6].

#### Progranulin is a ubiquitous, anti-inflammatory molecule

Progranulin participates in many biological functions, including development, inflammation, growth and cell motility. In the brain, it acts as a neuronal growth factor, regulates synaptic structure and function, and is a modulator of neuroinflammation. Progranulin is characterized by anti-inflammatory function that in part can be explained by its binding as a ligand for TNFR2 expressed by microglia [26] (Fig. 2). Studies in GRN-deficient mice have shown overproduction of TNF and MCP-1 and increased spontaneous, age-dependent activation of astrocytes and microglia. Furthermore, neurons from GRN-deficient mice were shown to be more susceptible to damage by activated microglia and depletion of oxygen and glucose [116]. GRN knockout mice have demonstrated excessive activation of microglia with abnormal phagocytosis and excess of proinflammatory cytokines rendering them neurotoxic [60, 92]. Accordingly, the loss-of-function mutation in GRN in humans is associated with dysregulation of pro-inflammatory cytokines, in particular IL-6 [116]. Progranulin is produced by a wide range of cell types, and in the CNS it is mainly released by mature neurons and microglia, with low levels found in astrocytes and endothelial cells [90]. The production of GRN in microglia and astrocytes is dependent on the state of activation whereas in neurons, GRN increases with age and is dependent on the neuronal activity associated with BDNF (brain-derived neurotrophic factor). Increased expression of GRN is associated with neurodegeneration [2, 75]. Strong upregulation of progranulin may occur in astrocytomas and associated vascular cells [52].

There are different mechanisms of GRN regulation in microglia as compared to astrocytes. In microglia the production is stimulated by anti-inflammatory cytokines such as IL-4 and IL-13 and inhibited by inflammatory stimuli such as LPS or IL-1β/IFN-γ (Fig. 2). Conversely, in astrocytes GRN is stimulated by inflammatory agents, including IL-1β/IFN-γ or the toll-like receptor 3 ligand and polyinosinic:polycytidylic acid (poly I:C) [90]. More importantly, astrocytes in contrast to microglia are a key source of secretory leukocyte protease inhibitor (SLPI), especially in humans [90]. GRN mutation carriers displaying the highest levels of SLP1 have later onset of disease [27]. SLPI inhibits proteolytic GRN cleavage caused by microglial MMP-12 which results in production of granulins, small molecules with pro-inflammatory properties. Granulins have been found in inflammatory CNS conditions including an animal model for spontaneous MS [62]. Thus, astrocytes might control and regulate microglia activation by secretion of SLP1 and the complex interplay among progranulin, MMP-12 and SLP1 may regulate inflammatory responses in vivo.

#### The pathology of GRN-FTLD involves gliosis and has a strong inflammatory component.

Astrocytosis and microgliosis with an abnormal lysosomal activity and activation of microglia with strongly enhanced phagocytic abilities as a result of chronic neuronal stress are characteristic for GRN-FTLD [96]. GRN haploinsufficiency is associated with abnormal microglial activation and neurodegeneration. Astrocytosis and microgliosis accompanying increased TDP-43 phosphorylation are especially evident in homozygous Grn knockout mice [45]. Large human GWAS data involving the immune system have provided evidence for broad genetic overlap between FTLD and immune-mediated disease genes, particularly in the HLA region rich in genes associated with microglial function [10]. New candidates that can modulate the FTLD gene such as *PGBD5, LRRK2 and TBKBP1* have been found. The latter two of these genes are upregulated during inflammatory responses and may also be involved in the regulation of TNF-α secretion. Importantly, elevated levels of TNF-α is a basic feature of FTLD [10]. It is worth noting that prolonged infectious or inflammatory conditions were observed in patients with FTLD decades before the onset of neuropsychiatric symptoms [116].

Potential treatments for GRN-FTLD patients might include GRN gene replacement, injected recombinant GRN protein or stimulation of GRN signaling. In a mouse model of FTLD, an overexpression of GRN resulted in normalized level of LAMP1 expression and a reduction in lysosomal abnormalities [5]. Several alkalizing agents including chloroquine already used in humans for other applications were found to upregulate GRN levels in lymphoblasts from GRN-FTLD patients and organotypic cortical slice cultures from mice deficient for GRN [13]. However, due to the carcinogenic and obesity-promoting properties of GRN, boosting production of this molecule can be a difficult task with associated risks [96]. Recent studies have revealed disease modifiers for GRN-related FTLD, including SORT1, RIPK1 and PSAP, that may represent new targets for disease-modifying therapies [109]. Clearly, further research in FTLD genetics, neuroimaging and fluid biomarkers for early detection will be required to enable possible intervention with novel drugs.

### Microglia in astrocytoma

**Fig. 3:**
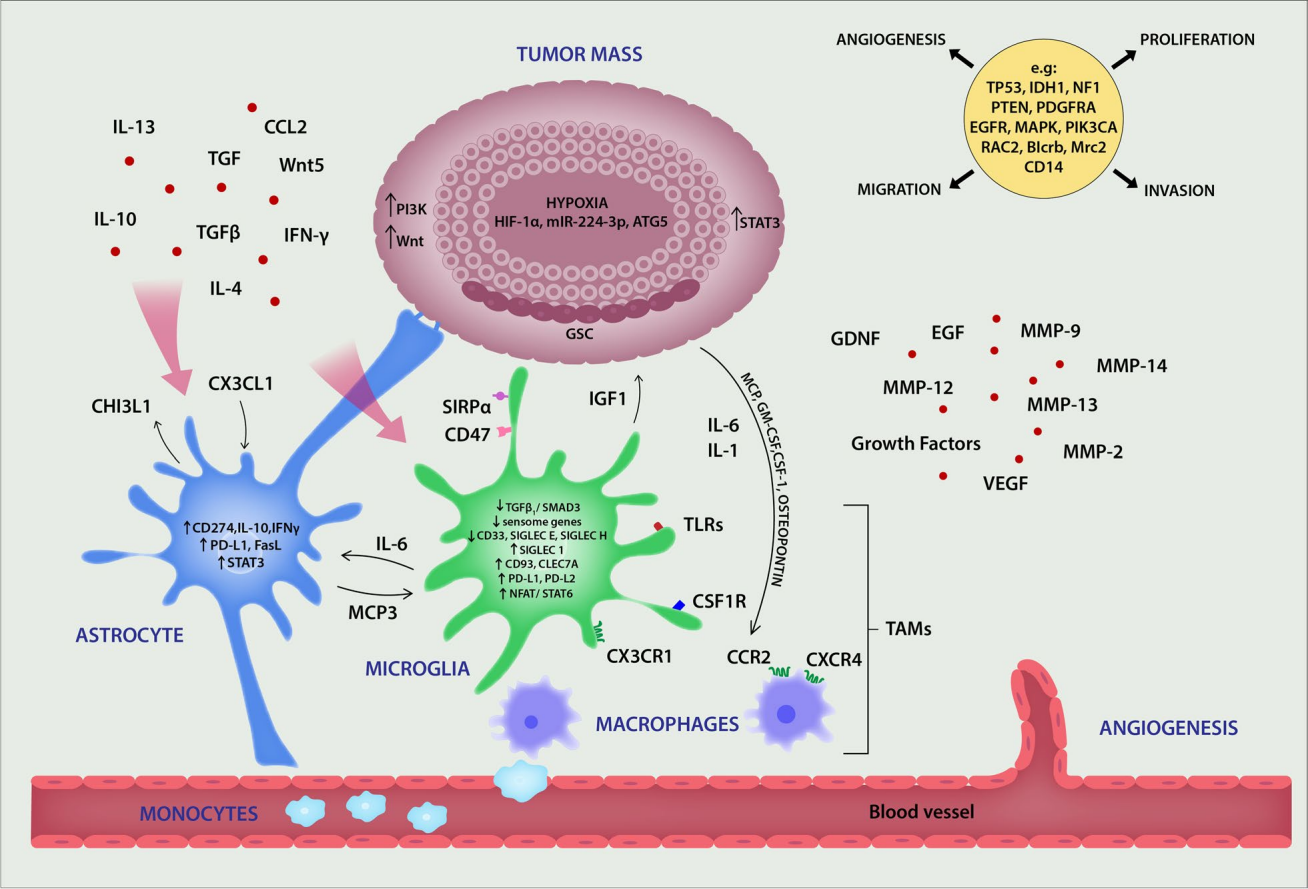
Participation of astrocytes, microglia and TAMs in glioblastoma-associated events. Active cross-talk among glia cells, macrophages and tumor cells via inflammatory modulators and receptors are taking place in astrocytoma. Activated glia and macrophages change their morphology and metabolism as well as undergo transcriptomic re-programming, actively participating in anti-tumor responses at the first stage of tumorigenesis. However, as tumor progresses, glia cells and TAMs reprogrammed by tumor milieu boost tumor growth and invasion. Some of molecules participating in the process are depicted. In the upper right corner some genes and molecular pathways participating in tumor proliferation, migration and invasion as well as an angiogenesis are presented. *ATG5* autophagy-related 5 gene, *CHI3L1* glycoprotein chitinase 3-like 1, *GSC* glioma-like stem cells, *HIF-1* hypoxia-inducible factor-1, *Wnt* wingless-type MMTV integration site family, *TAMs* tumor-associated macrophages

Astrocytoma is the most common primary brain tumor derived from astrocytes. Grade IV astrocytoma, called glioblastoma multiforme (GBM), is one of the most aggressive and deadly malignant tumors [114]. Like astrocytes, astrocytic tumors have marked heterogeneity, which would appear to be the main cause of poor treatment efficacy. A recent study by John Lin et al. using an intersectional, FACS-based approach allowed the identification of five distinct astrocyte subpopulations across three brain regions that show extensive molecular and functional diversity [42]. Some of the subpopulations possessed high proliferative and migratory properties typical of highly invasive brain tumors. It is therefore possible that malignant cells might originate from such populations. It was reported that certain adult brain subpopulations of astrocytes correlated with cellular populations present in highly heterogenous gliomas in mice and humans with increased expression of genes linked to synapse formation [42]. The high cellular heterogeneity with genetic and epigenetic variability are features of GBM [113]. Exome and transcriptome sequencing revealed a diverse array of recurrent genomic mutations in these tumors, including *TP53, IDH1*, *NF1, PTEN*, *PDGFRA*, *EGFR* and MAPK pathway mutations [102, 106]. During the development and progression of astrocytoma, there is a close interplay among neurons, astrocytes and microglial cells that promote tumor development, growth and invasion [25, 65, 78, 100] (Fig. 3). It is apparent that glioma tumor progression is facilitated by neuronal activity [101]. In turn, the activated cancer cells promote neuronal and synaptic activity [42]. Malfunction of the latter leads to seizures, which are a hallmark of glioma and tumor progression.

Normal cellular communication among brain cells is severely interrupted in astrocytomas. Cells lose their ability to communicate via calcium signaling, which becomes unsynchronized. The calcium signaling is mainly used by tumors for increased cell mobility and chemotaxis. However, targeting the calcium pathway would be non-specific, as it would affect both neurons and glial cells. Cross-talk among microglia and astrocytes with neoplastic cells is possible through different mechanisms of communication and transportation such as direct cell-to-cell contact, cytokines, chemokines, neurotropic factors, nanovesicles and non-vesicular mediated secretion [65]. In addition, almost 50 human ABC transporter proteins are involved in the active transport of a wide range of substances, including immune modulators, which participate in microglia/astrocyte/GBM intercommunication and some of these genes are known to be overexpressed in gliomas [8]. Targeting the microenvironment, especially glial cells within and around cancer tissue, has gained favor as a future therapeutic approach.

#### Activated TAMs and astrocytes in astrocytoma

Glia cells are first to react to the alterations in tumor microenvironment. Poor prognosis of astrocytoma correlates with an increased number of activated microglia and infiltrating macrophages. The tissue macrophage compartment in glioma or tumor-associated macrophages (TAMs), including resident microglia, accounts up to 30–50% of all cell types in gliomas, and the increase in their number correlates with a higher grade and worse prognosis. Resident microglia constitute a smaller portion, about 15% of TAMs, and reside in the tumor periphery whereas the rest are infiltrating bone marrow-derived macrophages that occupy the perivascular area [14]. These two populations have unique gene expression profiles and play different roles in tumorogenesis. The role of microglia at early stages of tumor transformation is still unclear.

It is postulated that at the beginning, microglia use their immune machinery to stop the malignant process, whereas during tumor progression activated microglia support the GBM [111]. One of the existing problems in studying the involvement of particular macrophage populations is the lack of specific phenotypic markers. A recently identified microglia-specific transcriptional regulator, Sall1, may be promising in future studies [12]. Activated microglia and macrophages play a central role in the delivery of growth factors and signaling molecules to nearby neurons and transformed astrocytes. In response, GBM facilitates recruitment of TAMs by release of MCP, GM-CSF, CSF-1 and osteopontin [83, 112].

Microglia promote the spread of astrocytoma by secretion of enzymes, such as MMP2, MMP9 and MMP14 which degrade the extracellular matrix and facilitate tissue invasion [59]. However, MMP inhibitors failed in clinical trials, but may have some application as prognostic biomarkers [80]. Several chemokines and chemokine receptors were implicated in the invasion process: CCL2, a member of the monocyte chemoattractant protein (MCP) chemokine family, plays an important role in mediating monocyte migration through its receptor CCR2. CCL2 produced by tumor cells stimulates microglial IL-6 production, which leads to tumor growth and invasiveness [20]. Low CCL2 expression in glioma patients has been associated with a significantly prolonged patient survival [14]. However, meta-analysis of the data from clinical trials using neutralizing monoclonal antibodies against CCL2 to block the CCL2–CCR2 axis, did not give positive results. A recent report showed that the CXCR4 antagonist, peptide R, affects not only the migration of myeloid cells, but also tumor cell metabolism and proliferation, and seems to have some therapeutic value [80].

Astrocytes, together with glial progenitors, serve as a cellular origin for malignant glioma [119]. The molecular pathways and their regulations are different in GBM and non-transformed reactive astrocytes that nevertheless actively participate in tumor development and progression [108]. Like microglia, activated astrocytes change their morphology and metabolism as well as undergo transcriptomic re-programming, actively participating in anti-tumor responses, tumor growth and invasion. Unlike microglia, astrocytes numerously occupy the peritumoral area and are also present at the tumor edge, thereby creating a peripheral hypoxia [54]. Mutual astrocyte–GBM cross-talk is evident and eventually works in favor of tumor growth. Mesenchymal transition and increased resistance to glioblastoma therapy has been linked to astrocyte reactivity [70]. A transcriptional shift of glioblastoma cells towards a mesenchymal phenotype followed by increased proliferation and migration is partially caused by a glycoprotein Chitinase 3-like 1 (CHI3L1, also termed YLK-40) released by tumor-associated reactive astrocytes [115]. Pro-angiogenic and metastatic activity of CHI3L1 has been efficiently blocked in vitro and in vivo in an animal model of glioblastoma multiforme by neutralizing antibody treatment, highlighting the potential benefit of this approach [23]. Additionally, astrocytes protect glioma cells from chemotherapy partly through creating gap junctions with GBM [117]. At the tumor site, both microglia and astrocytes contribute to a positive loop based on IL-6 production by microglia triggering astrocytes to release MCP-3, a chemokine attracting more microglial cells [39]. A three-party cross-talk is maintained via CX3CL1 released by astrocytes and neurons and its receptor, CX3CR1 that is only present on microglia. CX3CL1 produced by human glioma cells enhance cellular influx and increase MMP expression and tumor invasion [24]. Many studies have focused on Glioma-like Stem Cells (GSC) that are found in the marginal zone near microglia/macrophages. The latter promote high invasiveness of GSC by secretion of IL-6 in a TLR-4 dependent manner. Depletion of IL-6 in vivo resulted in inhibition of tumor growth and subsequent microglia infiltration [20]. However, GSC represent less than 20% of the tumor mass, while the majority of cells are undifferentiated glial cells. It is possible that tumor cells use existing programs and/or existing cells to change the environment and foster malignant growth. Such activation of the neuronal milieu may as a consequence induce proliferation of glioma cells [42].

#### Immunosuppression in astrocytoma

Immunosuppression is the hallmark of a growing tumor despite the increased infiltration of immunologically competent cells such as resident microglia and macrophages. Of note, other subpopulations of immune cells participate in immunosuppression and contribute to glioblastoma tumor progression [79]. Recent analysis of the gene expression profile of microglia in a mouse model of glioblastoma using RNA sequencing, confirmed microglial role in suppression of the adaptive immune response to the tumor, reduction of capacity to directly kill tumor cells and promotion of tumor cell invasion and proliferation [57]. Microglia are unable to see cancer cells due to decreased expression of MHCII [65]. In addition, the tumor environment reprograms microglia and macrophages to act as promoters of tumor growth and invasion in part by creating an anti-inflammatory milieu [35]. Cytokines such as IL-6, TGF-β, IL-10 and IL-4 dominate over inflammatory TNF-α, IL-12 and IL-2; and the NFkB signaling pathway stimulated by TNF-α is significantly downregulated in high-grade gliomas. Glioblastoma cells block astrocytic anti-tumor responses partially by releasing IL-10 and IFN-γ resulting in tumor growth [33, 66]. In response, tumor-reactive astrocytes increase expression of CD274 as well as IL-10 and IFN-γ by reprogramming myeloid cells and upregulating PD-L1 and FasL [37]. Moreover, IL-4, through activation of NFAT and STAT6 transcription factors, induces the expression of IGF-1 in TAMs, which signals neighboring tumor cells to activate the PI3K pathway to promote cell proliferation and tumor expansion.

Most glioblastoma patients show a hyperactive PI3K pathway due to either PTEN alterations or PIK3CA mutations [85]. Of interest, IGF-1 can increase activity of the PI3K pathway found in CSF-1R-resistant tumor cells. Combinations of IGF-1, NFAT or STAT6 inhibitors with CSF-1R inhibitors partially prevented tumor recurrence. Clinical trials focusing on inhibition of CSF-1R signaling, on which microglia/macrophage functions critically depend, have failed, even though the treatments enhanced phagocytosis. There is some hope in STAT3 blockade, which inhibits genes that stimulate the cell cycle and glioblastoma growth by blocking apoptosis. STAT3 is not normally activated in healthy brains under basic conditions. Conversely, the STAT3 pathway is highly activated in glioma and in turn inhibits activity of microglia and macrophages [80]. Several strategies targeting microglia/macrophages have been employed, including depletion, inhibition of recruitment and angiogenesis and enhancement of glioma invasion and immune potentiation. Thus far, none of these approaches were effective for treatment of glioblastomas.

Recently, particular attention has been paid to the Wingless-type MMTV integration site family (Wnt) of lipidated and glycosylated proteins that regulate many biological processes, including cell communication and lifelong immune regulation. These proteins are abnormally activated in gliomas partly due to several mutations (Matias et al. 2018a). How the aberrant Wnt signaling pathway effects each cell population involved in cancer progression has not yet been established, although Wnt molecules like Wnt5a and Wnt3a can be secreted not only by GSC but also activated microglia and astrocytes [48]. However, a recent study showed that high levels of Wnt5a in glioma were associated with upregulation of inflammatory processes and microglia activation and infiltration [19].

Some metabolic restrictions, such as low glucose levels, a low pH, hypoxia and the generation of suppressive metabolites facilitate immunosuppression and limit anticancer immune responses [18]. Hypoxia as a key feature of the tumor environment, especially in rapidly growing astrocytomas, and is the main factor in promoting tumor cell proliferation, invasion and drug resistance. Low oxygen pressure induces astrocytic secretion of CCL20 that further reinforces HIF-1α (hypoxia-inducible factor-1) in tumor cells [41]. It has been found that the HIF-1α/miR-224-3p/ATG5 pathway impacts the mobility of cells by regulating hypoxia-induced autophagy in glioblastomas and astrocytomas [38]. MiR‐224‐3p inhibits hypoxia-induced autophagy by directly targeting a key regulator of autophagy, an autophagy-related 5 (ATG5) gene that may be a novel target against hypoxia-induced autophagy in glioblastoma and astrocytoma. Hypoxic modification was found to influence both gene expression and metabolic changes that were associated with improved anticancer immune responses. Several metabolic modules have also been proposed for analysis during every phase of disease to help identify targeted, time-dependent therapies [105].

## Concluding remarks

**Fig. 4:**
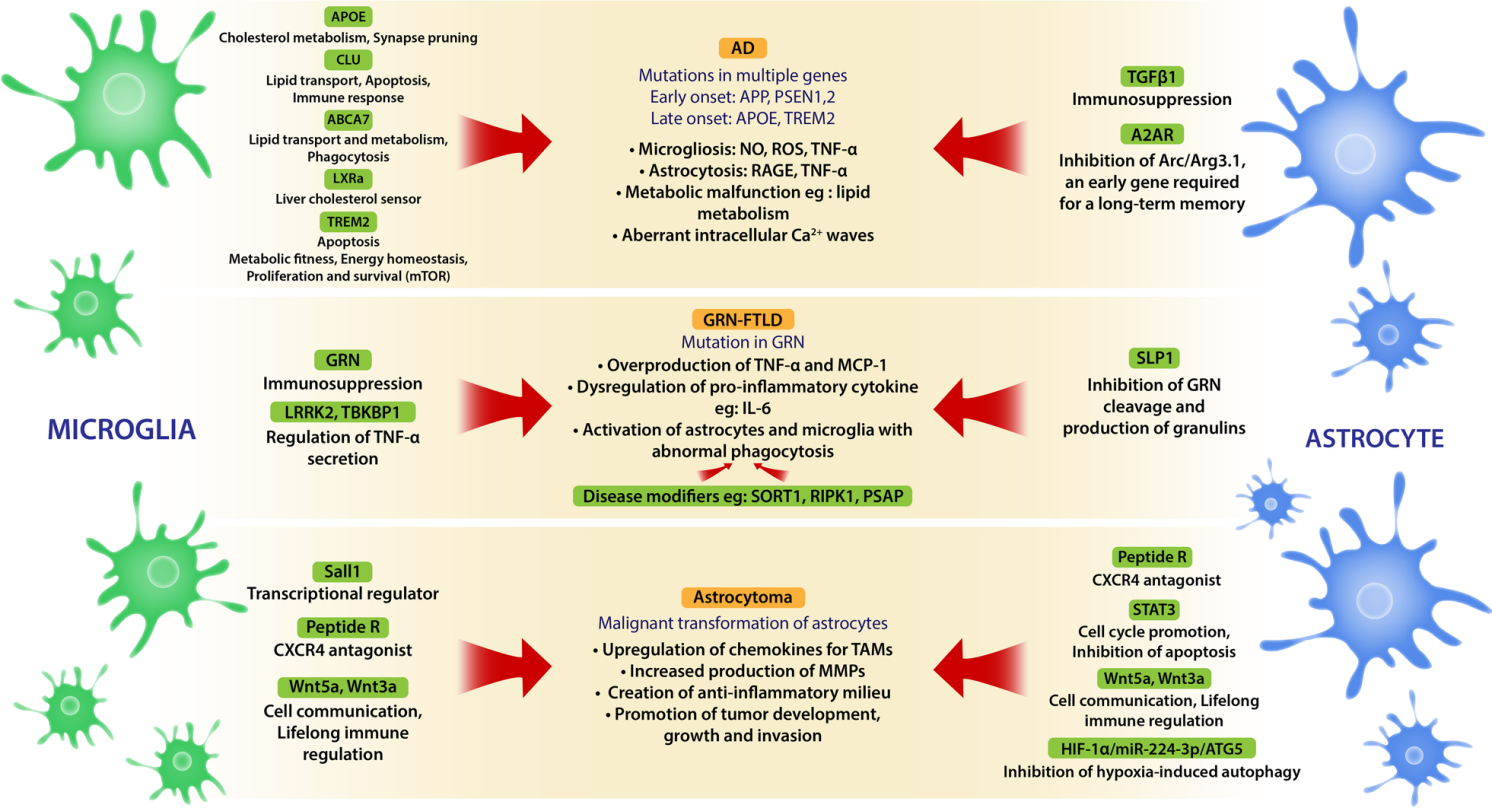
Key features, pathological processes and some candidates for therapeutic intervention in GRN-FTLD, Alzheimer disease and astrocytoma. Illustration presents some molecules expressed by microglia or astrocytes that modulate abnormal pathways activated in AD, GRN-FTLD and astrocytoma and therefore show some promise in treatment strategies. Among them are molecules that target metabolic malfunctions like lipid metabolism and energetic imbalance but also inflammatory factors contributing to neurodegeneration and factors involved in hypoxia, cell cycle, and lifelong immune regulation in cancer. *APOE* apolipoprotein E, *CLU* clusterin, *ABCA7* ATP-binding cassette subfamily A member 7, *LXRa* Liver X receptor alpha, *TREM2* triggering receptor expressed on myeloid cells 2, *TGFβ1* transforming growth factor beta 1, *A2AR* adenosine A2A receptor, *GRN* granulin, *LRRK2* leucine-rich repeat kinase 2, *TBKBP1* TBK1 binding protein 1, *SLPI* secretory leukocyte protease inhibitor, *SORT1* sortilin, *RIPK1* receptor-interacting serine/threonine-protein kinase 1, *PSAP* prosaposin, *Sall1* spalt like transcription factor 1, *Wnt* wingless-type MMTV integration site family, *STAT3* signal transducer and activator of transcription 3, *HIF-1a* hypoxia-inducible factor 1-alpha

Currently, the extraordinary complexity and heterogeneous nature of glia have become an obstacle in glia research [31]. Genetic studies highlight the prominent role for different subtypes of glia in susceptibility to different pathological disorders. Glia aging is also a key factor in the development of neuropathology. Genes induced by aging and involved in lipid and lysosomal biology are so far the only characteristics common for animal and human studies. Glial cells, especially astrocytes, are masters at providing metabolic fitness to the CNS, thus, targeting metabolic pathways may offer some therapeutic strategies. Recently, several different glial-specific targets, partially described in this paper, raise hope for discovering new therapeutics (Fig. 4). Special interest has been paid to the dysfunction of cellular metabolism and bioenergetic fitness as a possible genesis of neuropathology, for example the neuroenergetic hypothesis of AD or the Warburg and reverse Warburg hypotheses in cancer [98, 105]. Several different molecular glial targets can be modulated by gene therapy, recombinant proteins, epigenetic, transcription and translation regulators or nonsense suppression. Presently for AD and GRN-FTLD only symptomatic treatments are available. Currently although still not available, some disease modifiers known to upregulate GRN levels such as SORT1, RIPK1 and PSAP are being investigated [109]. Modifiers of age of onset are potential targets for disease-delaying therapies. There is a need for new tools, new techniques such as single-cell sequencing methods that produce unbiased, high-throughput data or different omics to grasp multiple functional and molecular patterns of glial cells that change upon environmental factors. Deciphering the nature of glial cellular diversity in the brain, how particular cell populations function and communicate in larger cellular networks during development, adulthood and aging and most importantly how to restore loss-of function in glia cells are the future goals for neurobiology.

## Data Availability

The datasets used and/or analyzed during the current study are available from the corresponding author on reasonable request.

## Abbreviations

ATP: Adenosine triphosphate
APP: AAβ precursor protein
ADE: Astrocyte-derived exosomes
AD: Alzheimer disease
AMP: Anti-microbial peptide
APOE: Apolipoprotein E
ABCA7: ATP-binding cassette transporter A7 (ABCA7—also a genetic risk factor for AD)
ATG5: Autophagy-related 5
Aβ: β-Amyloid
AMP: Anti-microbial peptide
BBB: Blood–brain barrier
CA^2^^+^: Calcium ion
CNS: Central nervous system
CCL2: Chemokine
CLU: Clusterin
CR: Complement receptors
IL-33: Cytokine
GBM: Glioblastoma multiforme
GDNF: Glial-derived neurotrophic factor
HIF-1α: Hypoxia-inducible factor-1
NFT: Intracellular neurofibrillary tangles
LXRa: Liver X receptor alpha
MHC: Major histocompatibility complex
MS: Multiple sclerosis
poly I:C: Polyinosinic:polycytidylic acid
PSEN1: Presenilin 1
PSEN2: Presenilin 2
GRN-FTLD: Progranulin-related frontotemporal dementia
RAGE: Receptor for advanced glycosylation receptors
SRs: Scavenger receptors
SLPI: Secretory leukocyte protease inhibitor
SIGLEC: Sialic acid-binding immunoglobulin-like lectin
TDP-43: Transactivating DNA binding protein
TREM2: Triggering receptor expressed on myeloid cells 2
TAMS: Tumor-associated macrophages
TNF: Tumor necrosis factor
Wnt: Wingless-type MMTV integration site family
